# Situational judgement testing at different stages of undergraduate medical training and the risk of professionalism lapses: A cohort study

**DOI:** 10.1111/medu.70020

**Published:** 2025-08-24

**Authors:** Gurvinder Sahota, Paul A. Tiffin, Daniel Smith, Edward Tyrrell, Mandy Hampshire, Jaspal Taggar

**Affiliations:** ^1^ Lead for Early Clinical and Professional Development, Clinical Associate Professor, School of Medicine University of Nottingham Nottingham UK; ^2^ Hull York Medical School University of York York UK; ^3^ School of Medicine University of Nottingham Nottingham UK

## Abstract

**Introduction:**

Identifying medical students at risk of professionalism lapses is critical for future patient safety. Situational Judgement Tests (SJTs) are commonly used to assess procedural knowledge of professionalism. Evidence suggests SJT scores can predict professionalism lapses, though longitudinal data across different stages of medical training are lacking. This study investigates the relationship between SJT performance at three points in pre‐qualification medical education and professionalism lapses.

**Methods:**

A longitudinal study was conducted using linked data from the UK Medical Education Database (UKMED) and the University of Nottingham. Data were available for 705 students who sat a professionalism‐focused SJT in their second year (2016 to 2018). Professionalism lapses were identified using concern forms. The study used scores from three SJTs administered at different stages: the University Clinical Aptitude Test (UCAT) at application; a Nottingham Medical School (NMS) test mid‐training, and the UK Foundation Programme (UKFP) SJT, at the end of medical school. The relationship between SJT scores and the odds of a professionalism lapse was modelled using logistic regression.

**Results:**

Performance on the NMS SJT was statistically significantly associated with the odds of professionalism lapses (Odds Ratio [OR] 0.64, p = 0.001) even after adjusting for educational and cognitive performance (OR 0.68, p = 0.005). The UKFP SJT scores showed a univariable (0.66, p = 0.006) but not an independent relationship with the outcome (OR 0.77, p = 0.14). The UCAT SJT scores were not statistically significantly predictive of professional lapses, on univariable (OR 0.85, p = 0.34) or multivariable analysis (OR 0.92, p = 0.66).

**Conclusions:**

SJTs can support medical professionalism in students. They are likely to be most effective in this context when they are administered during training, rather than as a selection measure for entry to medical school. Moreover, SJTs designed to have specific, professionalism‐focused content and a limited cognitive load are most likely to add unique value in this respect.

## Abstract 2

New study highlights the potential of SJTs beyond selection! Lower SJT scores may help identify students at risk of professionalism lapses, guiding targeted remediation.

## 1 INTRODUCTION

A condition of a licence to practice is that doctors demonstrate consistent professional behaviour.^1^ Regulators and other stakeholders, such as medical schools, have a duty to uphold standards of conduct in both students and doctors.^1^, ^2^, ^3^ This is required to ensure patient and public trust in the medical profession. The recruitment process for health professionals should therefore select on personal attributes relevant to consistent, professional, person‐centred care delivery. Educators can also help learners develop the interpersonal skills, amenable to training, relevant to ethical practice.^4^ Indeed, professionalism is a core topic within medical school curricula.

The construct of professionalism is complex. It can be conceptualised as occurring on two levels: a societal level, as a social contract between professions and society, and at an individual level.^5^ In this context, the ‘social contract’ aspect is most relevant; in return for practising privileges, society holds behavioural expectations of the profession's members. Thus, the public expects transgressions to be sanctioned. Underlying observable professional behaviours are ‘latent’ (not directly observable) traits and abilities. These are likely to include personality traits such as conscientiousness and agreeableness, as well as relevant aspects of ‘emotional intelligence’.^4^ ‘Values’ are also relevant, though again, not directly measurable. Values are attitudes and beliefs that determine individual goals and guide actions.^6^ Thus, there is no single method for measuring personal attributes relevant to professionalism.^7^ Nevertheless, Situational Judgement Tests (SJTs) have been increasingly introduced, internationally, to medical selection.^8^ The increased interest in SJTs for personnel selection was stimulated by conceptualising them as tools to measure implicit trait policies (ITPs).^9^ ITPs are mental models' individuals hold about effective workplace behaviours and are influenced by experience. SJTs can also, more simply, be considered as tests of procedural interpersonal knowledge about appropriate and effective behaviours.^10^ Such knowledge may be considered a necessary, though not sufficient, condition for exhibiting professionalism.^11^ The instructions in an SJT influence to what extent ITPs (which include attitudes) and/or procedural knowledge are measured; “What *would* you do?” question prompts tend to tap into the former, while “What *should* be done?” the latter.^12^

Performance on such SJT‐format assessments generally predicts professionalism lapses in both medical students and doctors.^13^, ^14^, ^15^ Importantly, prediction is often, though not always, incremental to that already offered by academic and cognitive metrics used in medical selection.^8^ In this context ‘professionalism lapses’ are behaviours that are recognised as contrary to established professional norms and mandated standards.^14^, ^16^ This includes a range of actions from ‘minor’ to those with implications for patient care and safety.^17^, ^18^, ^19^ Such misconduct is important to identify in students, and where possible, remediate as professionalism lapses during medical school predict subsequent malpractice post‐qualification.^20^, ^21^, ^22^

The ability of SJT scores to predict practice‐relevant overall behaviour may vary according to the career‐stage of administration; validity may be greater in post‐ vs pre‐qualification settings. This may be as pre‐medical school test‐takers and developers cannot draw on workplace‐specific content and experience.^8^ Moreover, the validity of SJTs is very sensitive to design factors, such as scoring systems.^23^ SJTs will detect changes in relevant procedural knowledge, interpersonal skills and attitudes in test‐takers. For example, changes in empathic abilities as clinical education progresses.^24^ Responses will also be influenced by the (‘anticipatory’, in the case of applicants) professional identity formation processes, in this respect students' core values shift, shaped by experiences and feedback.^25^, ^26^ Thus, SJTs are an opportunity to identify and remediate students lacking the appropriate knowledge and attitudes required to display consistent professionalism.

In the UK, there are potentially three SJTs medical students have taken: the University Clinical Aptitude Test (UCAT) SJT at medical school application, bespoke SJTs used for undergraduate educational purposes, and the UK Foundation Programme (UKFP) SJT formerly used towards graduation in the job allocation process. By linking these data, there is an opportunity to understand how performance on SJTs taken at different time points relates to professionalism in medical students. The aims of this study were therefore to determine the associations of SJTs with professionalism lapses across the undergraduate medical training pathway. The findings have implications for how such assessments are optimally used to enhance medical professionalism.

## 2 METHODS

### 2.1 Study design and data sources

A longitudinal study design was used, following a cohort of students at Nottingham Medical School (NMS) who had taken a professionalism SJT during the period from 2016 to 2018. NMS is a long‐established UK‐based medical school with an annual intake of approximately 240 students per year. During this time, NMS provided a mandatory, summative SJT‐format assessment as part of the second year studies. The medical school also has a system for reporting, recording and collating data on any professionalism lapses occurring during medical school studies (see below). These NMS data were then linked to national‐level data held in the UK Medical Education Database (UKMED).^27^ For the data linkage, one‐to‐one matching at the level of the medical student was performed by GMC‐employed staff using the individual's NMS internal identification number (also in the UKMED). This identifier was then replaced by a pseudo‐identifier code that cannot identify individuals prior to placing the data in the Safe Haven used by the researchers.^28^ Thus, the research team did not have access to identifiable data at any point.

## 3 DATA AVAILABILITY AND MANAGEMENT

### 3.1 Outcome

The primary outcome was any professionalism lapse occurring during medical school. At NMS, notification and management of professionalism concerns are recorded using a ‘concern form’.^14^, ^38^ This is advocated by institutions and medical regulators internationally.^38^, ^39^, ^40^, ^41^, ^42^ Concerns can be raised by staff and/or students using an electronic form submitted, recorded and collated on a central database. A free‐text section describes conduct concerns. The lead author (anonymised) reviewed the documented concerns against the GMC's Good Medical Practice standards, determining if they constituted a professionalism lapse. Lapses were also sub‐categorised according to the GMC‐specified domains of professional standards at that time (see Table 2).^44^ Given the sensitivity of these data, only the lead researcher (the NMS Early Years Professionalism Lead) was given access. Moreover, the data were pseudo‐anonymised, thus categorised blind to the student identity and SJT scores.

### 3.2 Predictor variables

The main predictors of interest were the three SJT scores. All three assessments were similar in conceptualisation and design, using text to describe situations and a selected‐response format for test‐taker answers. The use‐context, content, design and key psychometric properties of the SJTs are summarised in Table 1. Notably, all three SJTs were designed against a blueprint of the professional attributes relevant to exhibiting medical professionalism as described by the GMC.^45^ Reliability values cited for all the assessments were within the expected range for SJT‐format assessments.^14^, ^46^, ^47^ However, conventional metrics of reliability may be unsuitable for traditional SJTs.^48^

**TABLE 1:** Description of the design and key psychometric properties of the Situational Judgement Tests (SJTs) that generated the scores used in the study.

Situational Judgement Test (SJT)	Use context and development	Content and format	Psychometric properties
University Clinical Aptitude Test (UCAT) SJT	*Use context:* an SJT used as part of selection for the majority of medical and dental schools in the UK and some institutions in Australasia. The SJT component is used alongside the other cognitive subtests of the UCAT. *Development:* the UCAT SJT is text‐based and designed against a blueprint of which personal attributes medical and dental selectors felt were most relevant to successful study and a sustained career in the professions. The ‘critical incident technique’ (CIT) method was used to capture interpersonal situations via workshops. These situations were intended to elicit the relevant attributes in test‐takers. A scoring rubric was designed based on subject matter expert (SME) consensus regarding how appropriate or effective each depicted behaviour is perceived to be.	*Content:* depicts interpersonal scenarios related to everyday life and educational settings. The domain content relates to themes of “integrity”, “perspective taking”, “resilience” and “adaptability”. *Response format/s:* a rating scale format is used. This asks the test‐taker to rate the ‘appropriateness’ or ‘effectiveness’ of the portrayed actions, linked to the scenarios, on four‐point Likert‐format scales. *Length:* 69 SJT questions linked to around 22 scenarios. Candidates have 26 minutes to complete the SJT, though more time may be allowed for test‐takers with educational needs.	*Reliability:* Cronbach's alpha reported as around 0.81–0.86.^29^ An independent analysis, adjusting for dependency between items sharing a scenario, cited McDonald's Omega reliability values of 0.67 to 0.76.^30^ *Evidence for validity:* the SJT scores are statistically significantly correlated with subsequent tutor ratings relating to perceived ‘integrity’, ‘team‐working’ and ‘perspective taking’ in medical students.^31^ The scores predict subsequent misconduct, as indicated by ‘Fitness to Practice’ declarations, in medical students.^13^ This prediction is independent of scores on the cognitive component of the UCAT.
Nottingham Medical School (NMS) Professionalism SJT	*Use context:* an SJT used as a summative assessment at the end of second year at medical school. *Development:* the NMS SJT is text‐based and designed to assess knowledge relevant to the GMC Good Medical Practice standards. The ‘critical incident technique’ (CIT) method was used to capture pertinent interpersonal situations via workshops. A scoring rubric was designed based on subject matter expert (SME) consensus regarding how appropriate or effective each depicted behaviour was perceived to be.	*Content*: Scenarios and the associated responses depict behaviours relevant to the GMC domains of professionalism outcomes.^1^, ^32^ *Response format*: a mixture of rating and ranking response formats are used. The rating type questions ask test‐takers to rate the ‘appropriateness’ or ‘effectiveness’ of the portrayed behaviour on a five‐point Likert‐format scale. Best three answers format: Choose the three most appropriate responses from the 8 options available. Length: 21 separate SJT scenarios. 12 ranking style and 9 best three formats. Students had 120 minutes to complete the assessment.	*Reliability:* Cronbach's alpha reported as 0.52–0.54. *Evidence for validity:* Relatively low scores on the SJT were statistically significantly associated with having multiple professionalism lapses (>1) in medical students, independent of gender.^14^
UK Foundation Programme (UKFP) SJT	*Use context:* a national assessment used, until 2024, as part of the allocation process, deciding where newly qualified UK doctors would be placed for the Foundation Programme. The SJT scores were added to the Educational Performance Measure (EPM) to provide a total score to inform the decision‐making in the light of the individual preferences for placement locations expressed. *Development*: the ‘target’ attributes were decided by consultation with stakeholders. These were the personal qualities deemed most relevant to effective working as a Foundation Years doctor. Scenarios were developed in collaboration with SMEs from a range of medical specialties. ‘Item Development Interviews’, using the CIT, were conducted. SMEs participated in writing workshops and telephone interviews and that reflect the SJT target attributes. A scoring rubric was designed based on subject matter expert (SME). The SJT was mainly presented in text format. However, a small number of video‐format scenarios were included at times. Moreover, a few of the scenarios were designed to be linked via a theme that evolved over two or three situations.^33^	*Content:* The scenarios were described in clinical settings and were selected to relate to the personal attributes of ‘patient focus’, ‘commitment to professionalism’, ‘coping with pressure’, ‘effective communication’ and ‘teamwork’. *Response format:* a mixture of ranking, multiple choice and rating formats were used: Ranking‐ Candidates were asked to rank the 5 response options presented in order of their appropriateness or importance on a scale from one to five (e.g., 1 = Most appropriate; 5 = Least appropriate). Best three answers format ‐ Candidates were asked to select the three most appropriate response options, from the eight presented, which they felt would best resolve the presented situation. Rating‐ Candidates were asked to independently rate each of the 4–8 response options, in order of their perceived appropriateness or importance. *Length*: 75 scenarios were included of which 65 were operational and contributed to the candidate's score, while 10 were pilot scenarios. Candidates had 140 minutes to complete the SJT.	*Reliability*: Cronbach values cited as 0.84–0.85, though adjustments for dependency within items sharing scenarios were not made.^33^ *Evidence for validity*: the UKFP SJT scores show a statistically significant association with markers of professionalism^15^, ^34^ and conscientiousness.^35^ The scores statistically significantly predicted supervisor‐rated performance and incidence of remedial action in trainee physicians.^36^ They also statistically significantly predicted successful completion of the Foundation Programme, independent of EPM achievement.^37^

The national (UKFP and UCAT) SJT scores were standardised as z‐scores for each sitting. This facilitated comparisons across cohorts.^49^ While restriction of range can be a concern, empirical research demonstrating that restriction based on a predictor variable (SJT scores in this instance) allows correct estimation of regression coefficients whereas restriction based on an outcome variable (not a concern in this instance) does not. [Correction added on 19 December 2025, after the first online publication: The preceding sentence was corrected.] Similarly, standardising the NMS SJT score allowed (approximate) test equating across the three years.

### 3.3 Demographic and educational variables

From a patient and public safety perspective, it is the raw (unadjusted) relationship between the scores from SJTs used in high‐stakes settings, and the risk of misconduct that is most relevant. However, any observed associations could, at least partly, be mediated by demographic characteristics. Specifically, females^34^, ^50^, ^51^ and minority ethnic groups^34^, ^52^ may differ in both average SJT performance and proneness to disciplinary action. Thus, including such variables in analyses may shed light on the underlying reasons for any observed associations and have implications for the equitable and practical use of such tests. In this regard, a number of demographic variables and information on educational and cognitive performance were utilised via the UKMED. These were:

- Age at entry to medical school (years).
- Sex.
- Ethnicity (dichotomised into self‐identified as “White” or “ethnic minority”)
- Parental occupation (classified according to the UK National Statistics Socio‐economic classification (NSSEC) system. This was then dichotomised into ‘lower supervisory/technical and semi‐routine/routine’ occupations and other classifications associated with more advantaged socio‐economic background, as in previous research.^53^
- Nationality (primary nationality held‐ UK vs non‐UK).
- Disability (none vs some disability recorded).
- Cognitive performance (the UCAT total of the cognitive subtest scores, standardised as z‐scores according to all test‐takers at that testing cycle year).
- Academic ability (Educational Performance Measure (EPM‐ a metric of relative performance within a specific medical school, and total advanced qualification ‘tariff’ at application to medical school).

These covariates were also used to inform the multiple imputation process for any missing values.

### 3.4 Statistical analysis

The descriptive statistics for the study sample were produced. The degree of intercorrelation (Pearson's r) between the three SJT scores was evaluated. The cognitive load of each SJT was also estimated via the correlation observed with the UCAT cognitive total scores. Univariable logistic regression was used to model the (unadjusted) association between the SJT scores at each timepoint and the odds of professionalism lapses. To evaluate incremental validity, over and above academic achievement and cognitive ability, multivariable analyses were conducted adjusting for EPM, tariff score and UCAT cognitive total score. Finally, to understand the extent to which the predictive ability of the SJT scores were independent of any demographic or education factors, a separate set of multivariable models were built and tested. Here, an adaptive least absolute shrinkage and selection operator (LASSO) method was used to robustly select any covariates that significantly and independently predicted the outcome over and above each SJT score.^54^, ^55^ Each SJT score was entered into separate LASSO processes.

The missing data were not extensive (Table 2). However, to preserve information and reduce the bias risk, multiple imputation was used to create 30 imputed datasets for the logistic regression analyses using chained equations. The results from imputed and non‐imputed data were compared. Sensitivity analyses were also carried out in the form of subgroup analyses by sex and self‐identified ethnicity. All data management and analyses were conducted in the Trusted Research Environment (TRE) of the Health Informatic Centre hosted by the University of Dundee^28^ using Stata v17 MP.^56^

**TABLE 2:** Key demographic and educational characteristics of the study cohort and the number of individuals with professional lapses reported in each domain.

Characteristic	Blunted proportions (%)	Missing data blunted proportions (%)
**Sex** (assigned at birth)		1/705 (<1%)
Male	425/700 (60%)	
Female	280/700 (40%)	
**Ethnicity**		51/705 (7%)
White	400/650 (61%)	
Asian or Asian British	190/650 (29%)	
Black or Black British	15/650 (2%)	
Mixed	30/650 (4%)	
Other Ethnic Groups	20/650 (3%)	
**Parental Occupation**		195/705 (27%)
Semi‐routine and routine occupations	40/505 (8%)	
Lower supervisory and technical occupations	10/505 (2%)	
Small employers and own account workers	20/505 (4%)	
Intermediate occupations	40/505 (8%)	
Managerial and professional occupations	400/505 (78%)	
**Primary Nationality on Application**		0/705 (0%)
UK	580/705 (82%)	
Rest of the world	125/705 (18%)	
**Disability on entry to medical school**		0/705 (0%)
No known disability	650/705 (92%)	
Physical disability	20/705 (3%)	
Specific learning difficulty	20/705 (3%)	
Mental health condition or social communication/autistic spectrum disorder	10/705 (1%)	
	**Mean Z score (SD)**	**Proportion missing**
UCAT SJT score (z score)	0.35 (0.75)	85 (12%)
UK Foundation Programme (UKFP) SJT score (z score)	0.22 (0.84)	90 (13%)
**Professional lapses reported**		
**Category of lapse**	Number of individuals with lapses (%)	
1‐ Knowledge, skills and development	45/65 (69%)	
2‐ Patients, partnership and communication	20/65 (31%)	
3‐ Colleagues, culture and safety	5/65 (8%)	
4‐ Trust and professionalism	0/65 (<5%)	
Any lapse (≥1 lapse on any category)	65 (HESA blunted)	

* All values reported in Table 2 are rounded (blunted) in accordance with HESA statistical disclosure regulation, which includes blunting to 0 for values <3).^43^

### 3.5 Ethics

Ethical approval was granted by the University of Nottingham School of Medicine Research Ethics Committee [FMHS 103–0920]. Analyses of the UKMED database were approved by the institutional review board ethics exemption as they were undertaken in accordance with pre‐approved UKMED protocol.^57^, ^58^ Moreover, the study data were held in a TRE (‘safe‐haven’) where individual data cannot be extracted.^28^, ^59^ Moreover, any results from the UKMED must be presented in blunted form, with frequencies rounded to the nearest five.^43^

## 4 RESULTS

### 4.1 Sample characteristics

The sampling frame consisted of 720 students who sat the NMS SJT between 2016 and 2018. The SJT was mandatory, so the scores were available for all these students. Missing UCAT and UKFP SJT scores were predominantly due to some international students not sitting these assessments. A small number of overseas elective students (n = 35) were excluded from the sampling frame. Moreover, 15 students were recorded as having entered medical school prior to 2014. These observations were excluded from the analytic sample to maintain a consistent ‘exposure time’ (the period during which professionalism lapses could be reported). The participant characteristics are described in Table 2. Overall, 65 (HESA blunted) professional lapses were recorded, with most being in the domain of ‘knowledge, skills and development’ (Table 2).

Post‐hoc power calculations indicated that, given the number of students and lapses observed, the study had 80% power to show an effect size represented by an Odds Ratio (OR) of 0.808 (or lower‐ i.e. larger effect size).

### 4.2 Univariable results

The three study SJTs scores showed statistically significant, albeit small, intercorrelations: UCAT with NMS SJT, r = 0.16; NMS with UKFP SJT, r = 0.25; UCAT with UKFPO SJT, r = 0.23; associated p values <0.0001 in all cases. The scores from the UKFP SJT showed the highest degree of correlation with the UCAT cognitive total score (r = 0.26) and the NMS SJT the least (r = 0.11). The UCAT SJT scores correlated 0.23 with those from the cognitive component of the assessment (p < 0.0001 in all cases). The results for the univariable analyses for the association between the SJT scores and the odds of at least one professionalism lapse are presented in Table 3. The largest effect size was observed for the NMS SJT scores (OR 0.64, 95% confidence intervals [CIs] 0.50 to 0.83, p = 0.001). This result can be interpreted as follows: for every SD above the mean for their year group that was scored on the NMS SJT, the odds of a professionalism lapse reduced, on average, by 36%. This interpretation holds approximately in the middle of the distribution of scores, given the non‐linear nature of odds ratios. The relationship was also statistically significant for the NMS SJT scores predicting lapses in two specific domains of professionalism (‘*knowledge …* ’ and ‘*trust … ’*‐ see Table 3). The univariable (raw) effect‐size was similar for the UKFP SJT scores (OR 0.66, CIs 0.49 to 0.89, p = 0.006) and was also statistically significant for lapses in one specific domain (‘*colleagues …*’‐ see Table 3). In contrast, a more modest, non‐statistically significant effect size was observed for the UCAT SJT scores (OR 0.85, CIs 0.60 to 1.19, p = 0.34). The relationship between the UCAT SJT score and lapses specifically in the domain of ‘*colleagues, culture and safety*’ was of borderline statistical significance (OR 0.64, 0.40 to 1.02, p = 0.06).

**TABLE 3:** Results from univariable and multivariable logistic regression analyses predicting the odds of a professionalism lapse from the standardised SJT scores in the multiply imputed data (m = 30).

	UKCAT SJT		Nottingham SJT		UKFPO SJT	
	Odds Ratio (OR) (95% CI)	p‐value	Odds Ratio (OR) (95% CI)	p‐value	Odds Ratio (OR) (95% CI)	p‐value
*Univariable analyses*						
Any lapse	0.85 (0.60–1.19)	0.340	0.64 (0.50–0.83)	0.001	0.66 (0.49–0.89)	0.006
Knowledge, skills and development	1.10 (0.68–1.78)	0.696	0.53 (0.37–0.76)	0.001	0.70 (0.46–1.05)	0.082
Patients, partnership and communication	0.58 (0.14–2.31)	0.438	0.31 (0.11–0.88)	0.028	1.10 (0.27–4.25)	0.919
Colleagues, culture and safety	0.64 (0.40–1.02)	0.063	0.72 (0.506–1.028)	0.072	0.62 (0.42–0.93)	0.20
Maintaining trust	0.78 (0.45–1.35)	0.383	0.59 (0.40–0.87)	0.008	0.78 (0.49–1.24)	0.286
***Multivariable analyses*** *All models a*djusted for academic achievement (EPM and advanced qualification tariffs at application) and cognitive performance (UCAT cognitive total score)						
Any lapse	0.92 (0.64–1.33)	0.655	0.68 (0.52–0.89)	0.005	0.77 (0.55–1.08)	0.136
Knowledge, skills and development	1.30 (0.78–2.16)	0.316	0.58 (0.40–0.84)	0.004	0.86 (0.53–1.39)	0.536
Patients, partnership and communication	0.90 (0.19–4.32)	0.893	0.21 (0.05–0.89)	0.031	1.85 (0.31–11.08)	0.498
Colleagues, culture and safety	0.74 (0.44–1.22)	0.236	0.80 (0.55–1.17)	0.253	0.83 (0.52–1.33)	0.441
Maintaining trust	0.74 (0.41–1.32)	0.305	0.55 (0.36–0.83)	0.005	0.72 (0.43–1.22)	0.219
***Multivariable analyses*** adjusted for academic achievement (EPM) and male sex*						
Any lapse	0.98 (0.68–1.40)	0.912	Not applicable: for NMS SJT as no statistically significant confounding variables were identified by LASSO		0.79 (0.57–1.10)	0.165
Knowledge, skills and development	1.31 (0.79–2.16)	0.299			0.86 (0.54–1.37)	0.518
Patients, partnership and communication	0.62 (0.14–2.16)	0.517			1.04 (0.22–4.97)	0.953
Colleagues, culture and safety	0.75 (0.46–1.22)	0.243			0.78 (0.49–1.23)	0.283
Maintaining trust	0.83 (0.48–1.46)	0.526			0.771 (0.46–1.29)	0.324

* LASSO used to identify which demographic and educational variables should be retained in the multivariable models. For the UKFP and UCAT SJT, these were EPM and male sex. No covariates were retained for the NMS SJT multivariable model.

### 4.3 Multivariable results

The only SJT where the scores that remained statistically significant and independent predictors of professionalism lapse, after adjusting for academic and cognitive performance, were those for the NMS SJT (OR 0.68, CIs 0.52 to 0.89, p = 0.005). Moreover, even after just adjustment, the NMS SJT scores predicted lapses in three of the four specific professionalism domains (Table 3).

According to the LASSO procedure, EPM and sex were identified as potentially statistically significant predictors of professionalism lapse, independent of the UKFP and UCAT SJT scores. When these were entered into multivariable models, the respective SJT scores were no longer statistically significant, independent predictors of professionalism lapse (Table 3). However, LASSO did not identify any potentially statistically significant and independent predictors of professionalism lapse for the NMS SJT scores. Thus, such a multivariable model with additional demographic and/or educational covariates was not applicable in the case of the NMS SJT.

### 4.4 Results from sensitivity analyses

The results from logistic regression analyses of the non‐imputed data are shown in Table S1 of the Supplementary Material. As can be seen, the results do not vary substantially compared to those derived from the imputed data. The results of the subgroup analyses for the prediction of the odds of any professionalism lapse, according to sex and self‐identified ethnicity, are shown in Table S2 of the Supplementary Material. Likewise, as can be seen, allowing for the reduced study power, the pattern of associations between SJT scores and lapses is very similar to those observed for the sample as a whole.

An additional sensitivity analysis was conducted in relation to the UCAT SJT to explore potential reasons for the non‐significant associations with lapses. The UCAT was implemented in medical selection 2013, and existing studies report the predictive validity from these early data.^13^, ^31^ It may be that the increased familiarity with the test content via practice materials has influenced the validity of the scores, since. Thus, we compared the results for the UCAT SJT for 2013 to later years of sittings. These showed that the effect‐size for the 2013 UCAT SJT scores were somewhat larger than for subsequent years (ORs 0.72 vs 0.91, respectively; see Table S3 of the Supplementary Material).

## 5 DISCUSSION

To our knowledge, this is the first study to evaluate the ability of SJT scores, from different stages of undergraduate medical training, to predict lapses in student professionalism. In this respect, only the NMS SJT scores, derived mid‐training, were statistically significant, independent predictors of professionalism lapses. The UKFP SJT scores were not independent of academic and cognitive performance. Moreover, the effect sizes for the UCAT SJT scores were more modest than the other SJTs and did not reach statistical significance. There were no obvious patterns observed in relation to the SJT scores and lapses in specific domains of professionalism. The degree of intercorrelation between the SJT scores was relatively modest. This could be due to the differences in design and content of the SJTs, despite the broad similarities (Table 1), as well as the imperfect reliability inherent in psychological assessments.

Our results echo those from a previous study of the NMS SJT, which observed a similar magnitude of inverse relationship associations between higher scores and the risk of professionalism lapses in medical students.^14^ The sample in this latter study was smaller than the present one (N = 247) but detected a statistically significant relationship between the NMS SJT scores and the risk of multiple lapses, independent of sex. Here, we were able to show that the NMS SJT scores are statistically significantly predictive of professionalism lapse risk, even after adjusting for educational and cognitive performance. The relatively strong predictive validity of the NMS SJT scores may be partly explained by its content being closely mapped to the curriculum on professionalism at NMS. Thus, it may have tapped into conscientiousness, a key facet of professionalism, to a greater degree than the other SJTs. Furthermore, the cognitive loading of the NMS was less than half of that observed for the other two SJTS, as evidenced by the low (r = 0.11) correlation with the UCAT cognitive scores. This may have accounted for the incremental predictive validity observed.

The UKFP SJT scores have previously been reported to be significantly correlated with markers of conscientiousness.^35^ They also predict successful completion of the Foundation Programme, independent of EPM achievement.^37^ Of most direct relevance to this study, the scores have been shown to predict the risk of disciplinary action in qualified doctors.^15^ However, this latter study was insufficiently powered to estimate whether the observed relationship was independent of academic attainment.^60^ Nevertheless, it is clear from the present and previous study that the ability of the UKFP SJT to predict professionalism lapses is at least partly mediated by academic and/or cognitive performance.

The lack of statistically significant associations observed between the UCAT SJT scores and the risk of professionalism lapses contrasts with the findings of a similar, national‐level study.^13^ In this latter study, incremental validity, above that provided by the cognitive scores for the UCAT, was reported for predicting formal sanctions in medical students. Indeed, somewhat larger effect sizes were reported by Tiffin et al compared to those observed in the present study (e.g. OR 0.77 vs 0.92 for adjusted models). Two factors may explain these differences. Firstly, the outcome in the national study was at least one sanction in relation to professionalism lapses reported to the regulator by medical schools, in an annual return. Thus, the threshold for recording these would have been higher than for the NMS concern forms. Secondly, the national study used UCAT SJT scores from its introductory year. Coaching and practice effects can increase such SJT scores by around 0.5 of a standard deviation.^61^ Thus, these practice effects may have, at least modestly, attenuated the predictive validity of the UCAT SJT scores, in this respect. This possibility is supported by the relevant results of our sensitivity analyses (Table S3, Supplementary Material). Moreover, the UCAT SJT was administered prior to medical school. Thus, there was little or no relevant clinical workplace experience to draw on, both for test designers and test‐takers, explaining the generally smaller validity coefficients reported for SJTs administered pre‐ rather than post medical school.^8^

### 5.1 Strengths and Limitations

The longitudinal nature of our data was unique, and the size of cohort provided adequate study power to detect a moderate effect size on univariable analysis (i.e. an OR of around 0.8 or lower). However, there would have been insufficient power to detect more modest, and/or adjusted effects. Thus, we cannot rule out that at least some, our null results were due to type II errors. Nevertheless, if the effects, raw or adjusted, were smaller than our study was powered to detect, then it could be argued that they are of uncertain educational significance. Our ability to control for the effects of academic and cognitive performance also strengthened our study design. This meant we were able to estimate the incremental (unique) value that the SJT scores made in an educational context. Nevertheless, the single‐institution nature of our study means some caution should be exercised in generalising the results to other settings. However, the findings were broadly in line with those from national and meta‐analytic studies (see earlier).

Given the sensitive nature of the data, external review of the domain categorisation of the lapses was not possible. However, in this context, the fact that any lapse occurred is more critical than which domain was affected. Also, whilst missing data were not extensive, they were dealt with via multiple imputation, reducing the risk of bias. Moreover, the results from the imputed and non‐imputed data were almost identical. This provides evidence to support the ‘missing at random’ (MAR) assumption underpinning multiple imputation, i.e. that the relationship between the variables with observed values is the same as for those that are missing.

As with any reporting process, the identification of students with professionalism issues could be a source of bias. However, the concern form process was standardised and against set GMC standards, reducing the potential for biases. Moreover, the students were observed and supervised more closely compared to the doctors. Thus, it could be argued that any reporting bias was likely to be less marked than for studies reporting on professionalism lapses post‐qualification.

### 5.2 Implications for policy and practice

Our findings support the view that these ‘traditionally’ designed SJT‐format assessments are more effective as developmental and selection tools during, rather than before, medical training for admission. The validity of in‐programme SJTs may stem from their ability to tap into relevant procedural knowledge gained from teaching and/or clinical experience. Moreover, SJTs in this capacity may act as a proxy measure for professional identity formation, as students internalise the desirable values through socialisation, role modelling and clinical exposure. In this sense, formative SJTs with feedback could be conceptualised as a form of ‘scenario‐based learning’.^62^

Conversely, the UCAT SJT, widely used for medical school admission, showed no significant association with subsequent professionalism lapses. As previously argued, it may be more appropriate to use ‘construct‐driven’ SJTs, or other tools, that tap into more generic, desirable personal qualities in this context.^8^ However, at present, such assessments may behave more like personality self‐report tests, and their validity for personnel selection is, as yet not established.^10^, ^63^, ^64^ Thus, regulatory bodies and test developers, should re‐evaluate and potentially redesign pre‐admission SJTs. Conversely, the evidence for the use of such SJTs in later high‐stakes settings seems firmer. In this regard, the recent removal of the UKFP SJT from the postgraduate allocation process is a cause for concern; the scores from this tool predict unprofessional behaviour. It therefore may provide some level of public protection, and policymakers should consider reinstating such an SJT prior to medical qualification.

In line with previous findings, sex and educational performance were identified as significant ‘confounders’ for two of the SJTs, though notably not the NMS SJT. In terms of sex, this has less implications for SJT design; if females are higher on the traits relevant to professionalism, then their sex is not relevant to the selection process. Nevertheless, SJTs should still be evaluated for “differential item functioning” (DIF) by sex (and other demographic characteristics) in relation to equity. That is, certain items should not show significant bias for construct‐irrelevant qualities, such as sex. Regarding educational performance, care should be taken to reduce the cognitive load of SJTs to help ensure that the scores offer incremental values above academic and/or cognitive abilities. This seemed to be the case for the NMS SJT scores.

Educators can use any insights gained from mid‐course SJT performance, particularly those administered around key clinical transitions, to identify students who may benefit from additional support, allowing for early, targeted remediation. Indeed, emerging evidence suggests that structured remediation programs may enhance student and physician professionalism.^65^ Moreover, by using SJTs as formative, rather than summative assessments, educators can provide feedback that encourages student reflection and strengthens their commitment to professional development.

### 5.3 Directions for Future Research

Ideally, our findings would be replicated in a larger, multi‐site study, should an NMS‐style SJT be adopted at other institutions. Also, as SJT content becomes increasingly familiar or publicised, the validity of such instruments should be periodically evaluated. Our findings also provide fresh impetus to develop alternatives to traditional SJTs, capable of measuring key aspects of emotional intelligence relevant to healthcare in those lacking relevant workplace experience.^4^, ^10^, ^64^ Plausible candidate assessments could include generic tests of interpersonal understanding, such as the Empathic Agent Paradigm Test (EAPT).^66^ Importantly, it is crucial to establish the extent to which students flagged by low SJT scores could be remediated, and if this related to the specific domain of any lapse.

## 6 CONCLUSIONS

SJT scores can predict professionalism lapses in medical students, though the degree to which they can do this, independently of educational‐related metrics, varies according to the timing of administration. SJTs designed with professionalism‐specific content and a low cognitive load are those most likely to demonstrate incremental predictive validity over existing academic metrics. Our findings cast some doubt on the usefulness of traditionally designed SJTs for selection into medical school, and alternative tools should be developed and evaluated. Conversely, they suggest that the integration of such assessments, formatively, into medical curricula can support the development of professionalism in students. Such tests also have an important ‘gatekeeper role’ in post‐qualification medical selection and regulation. Further research should investigate if students flagged via low SJT scores can be successfully remediated.

## AUTHOR CONTRIBUTIONS

**Gurvinder Sahota:** Conceptualisation; methodology, visualisation; writing original draft, writing—review and editing (lead). **Paul A. Tiffin:** Methodology; formal analysis; writing—review and editing. **Daniel Smith:** Formal analysis; writing—review and editing. **Edward Tyrrell:** Methodology; writing—review and editing. **Mandy Hampshire:** Writing—review and editing. **Jaspal Taggar:** Conceptualisation; methodology; writing—review and editing; supervision.

## CONFLICTS OF INTEREST STATEMENT

The authors have no conflicts of interest to declare.

## Supporting information


**Table S1.** Results of the univariable and multivariable logistic regression analyses predicting the odds of a professionalism lapse from the standardised SJT scores from the non‐imputed data.
**Table S2.** Results of the subgroup analyses, according to male sex and self‐identified (non‐White) ethnicity in the imputed data (m = 30).
**Table S3.** Sensitivity analysis results comparing the ability of the UCAT SJT scores to predict a student with at least one professionalism lapse for the 2013 test compared to later rounds. Results from the imputed (m = 30) and non‐imputed data are shown.

## Data Availability

The data that support the findings of this study are available via the United Kingdom Medical Education Database (UKMED).
