# Supplementary material for: Situational judgement testing at different stages of undergraduate medical training and the risk of professionalism lapses: A cohort study
Source: Med Educ. 2025 Aug 24;60(3):296–306. doi: 10.1111/medu.70020 (PMC12913242; doi:10.1111/medu.70020)
Supplement: Supplementary file 1 — Table S1. Results of the univariable and multivariable logistic regression analyses predicting the odds of a professionalism lapse from the standardised SJT scores from the non‐imputed data. Table S2. Results of the subgroup analyses, according to male sex and self‐identified (non‐White) ethnicity in the imputed data (m = 30). Table S3. Sensitivity analysis results comparing the ability of the UCAT SJT scores to predict a student with at least one professionalism lapse for the 2013 test compared to later rounds. Results from the imputed (m = 30) and non‐imputed data are shown. [file MEDU-60-296-s001.docx]

**Supplementary Material for: Situational judgment testing at different stages of undergraduate medical training and the risk of professionalism lapses: a cohort study**

Gurvinder Sahota, Paul A Tiffin, Daniel Smith, Edward Tyrrell, Mandy Hampshire, Jaspal Taggar

This *Supplementary Material* document contains the results of the sensitivity analyses conducted in relation to the above study. The findings from these are relevant to making inferences from the results presented in the main report.

**Results from sensitivity analyses**

The results from logistic regression analyses of the non-imputed data are shown in Table S1 of this Supplementary Material. As can be seen the results do not vary substantially from those for the imputed data (m=30). For example, the relationship between the UCAT SJT scores and odds of a professionalism lapse in the domain of ‘*colleagues, culture and safety’*, remains of borderline statistical significance, albeit the p value has reduced slightly from 0.06 to 0.05.

**Table S1.** Results of the univariable and multivariable logistic regression analyses predicting the odds of a professionalism lapse from the standardised SJT scores from the non-imputed data.

|  | **UKCAT SJT** | | **Nottingham SJT** | | **UKFPO SJT** | |
| --- | --- | --- | --- | --- | --- | --- |
|  | Odds Ratio (OR) (95% CI) | p-value | Odds Ratio (OR) (95% CI) | p-value | Odds Ratio (OR) (95% CI) | p-value |
| ***Univariable analyses*** | | | | | | |
| Any lapse | 0.83 (0.58-1.18) | 0.290 | 0.64 (0.50-0.83) | 0.001 | 0.62 (0.46-0.84) | 0.002 |
| Knowledge, skills and development | 1.10 (0.67-1.81) | 0.712 | 0.54 (0.37-0.76) | 0.001 | 0.66 (0.43-1.00) | 0.048 |
| Patients, partnership and communication | 0.50 (0.12-2.12) | 0.346 | 0.31 (0.11-0.88) | 0.028 | 1.22 (0.30-5.00) | 0.779 |
| Colleagues, culture and safety | 0.62 (0.38-1.01) | 0.056 | 0.72 (0.51-1.03) | 0.072 | 0.58 (0.39-0.87) | 0.008 |
| Maintaining trust | 0.75 (0.42-1.32) | 0.314 | 0.59 (0.40-0.87) | 0.008 | 0.76 (0.49-1.19) | 0.229 |
| ***Multivariable analyses***  *A*djusted for academic achievement (EPM and advanced qualification tariffs at application) and cognitive performance (UCAT cognitive total score) | | | | | | |
| Any lapse | 0.75 (0.50-1.12) | 0.162 | 0.66 (0.49-0.89) | 0.006 | 0.73 (0.2-1.03) | 0.074 |
| Knowledge, skills and development | 1.06 (0.58-1.94) | 0.854 | 0.54 (0.35-0.83) | 0.005 | 0.80 (0.48-1.33) | 0.391 |
| Patients, partnership and communication | 1.16 (0.15-8.78) | 0.885 | 0.24 (0.56-1.02) | 0.053 | 2.33 (0.42-13.00) | 0.332 |
| Colleagues, culture and safety | 0.57 (0.32-1.01) | 0.053 | 0.86 (0.57-1.30) | 0.482 | 0.78 (0.48-1.27) | 0.320 |
| Maintaining trust | 0.67 (0.35-1.27) | 0.217 | 0.57 (0.37-0.90) | 0.015 | 0.71 (0.42-1.20) | 0.189 |
| ***Multivariable analyses***  *A*djusted for academic achievement (EPM) and male gender* | | | | | | |
| Any lapse | 0.87 (0.60-1.27) | 0.483 | Not applicable: for the NMS SJT scores no statistically significant confounding variables were identified by LASSO in relation to professionalism lapse prediction | | 0.76 (0.55-1.06) | 0.107 |
| Knowledge, skills and development | 1.15 (0.67-1.98) | 0.610 |  |  | 0.84 (0.52-1.33) | 0.451 |
| Patients, partnership and communication | 0.47 (0.10-2.13) | 0.328 |  |  | 1.50 (0.30-7.34) | 0.615 |
| Colleagues, culture and safety | 0.62 (0.37-1.03) | 0.067 |  |  | 0.77 (0.50-1.20) | 0.250 |
| Maintaining trust | 0.77 (0.43-1.40) | 0.394 |  |  | 0.78 (0.47-1.30) | 0.341 |

*LASSO used to identify which demographic and educational variables should be retained in the multivariable models. For the UKFP and UCAT SJT these were EPM and male sex. No covariates were retained for the NMS SJT multivariable model.

The results of the subgroup analyses, for the prediction of the odds of any professionalism lapse, according to sex and self-identified ethnicity are shown in Table S2. Due to the smaller number of observations, and hence reduced study power, these results must be interpreted cautiously. However, as can be seen, the pattern of associations between SJT scores and lapses, is very similar to those observed for the sample as a whole.

**Table S2.** Results of the subgroup analyses, according to male sex and self-identified (non-White) ethnicity in the imputed data (m=30).

|  | **UKCAT SJT** | | **Nottingham SJT** | | **UKFPO SJT** | |
| --- | --- | --- | --- | --- | --- | --- |
|  | Odds Ratio (OR) (95% CI) | p-value | Odds Ratio (OR) (95% CI) | p-value | Odds Ratio (OR) (95% CI) | p-value |
| **Results for males only** (n=275 [HESA rounded]) | | | | | | |
| ***Univariable analyses*** | | | | | | |
| Any lapse | 0.92 (0.56-1.47) | 0.729 | 0.62 (0.43-0.88) | 0.008 | 0.73 (0.49-1.08) | 0.116 |
| ***Multivariable analyses***  *A*djusted for academic achievement (EPM and advanced qualification tariffs at application) and cognitive performance (UCAT cognitive total score) | | | | | | |
| Any lapse | 0.96 (0.59-1.58) | 0.883 | 0.65 (0.45-0.94) | 0.021 | 0.82 (0.53- 1.28) | 0.384 |
| ***Multivariable analyses***  *A*djusted for academic achievement (EPM) only (sex does not vary in this subsample)* | | | | | | |
| Any lapse | 0.95 (0.59 to 1.52) | 0.833 | Not applicable | | 0.80 (0.52 to 1.24) | 0.322 |
|  |  |  |  |  |  |  |
| **Results for those identifying as of non-White ethnicity only** (n=255 [HESA rounded]) | | | | | | |
| ***Univariable analyses*** | | | | | | |
| Any lapse | 0.92 (0.54- 1.56) | 0.751 | 0.57 (0.38- 0.86) | 0.007 | 0.60 (0.39- 0.95) | 0.028 |
| ***Multivariable analyses***  *A*djusted for academic achievement (EPM and advanced qualification tariffs at application) and cognitive performance (UCAT cognitive total score) | | | | | | |
| Any lapse | 1.01 (0.57 to 1.80) | 0.974 | 0.62 (0.41 to 0.95) | 0.028 | 0.73 (0.45 to 1.18) | 0.199 |
| ***Multivariable analyses***  *A*djusted for academic achievement (EPM) and male gender* | | | | | | |
| Any lapse | 1.02 (0.58 to 1.78) | 0.952 | Not applicable | | 0.71 (0.44 to 1.13) | 0.151 |
|  |  |  |  |  |  |  |

*LASSO used to identify which demographic and educational variables should be retained in the multivariable models. For the UKFP and UCAT SJT these were EPM and male sex. No covariates were retained for the NMS SJT multivariable model.

As the results in relation to the UCAT SJT scores were observed to not reach statistical significance an additional sensitivity analysis was conducted. The UCAT SJT was piloted in 2012 and implemented in medical selection 2013. The studies reporting the predictive validity of the test currently only use data from its initial introduction. Since this time access to practice material and coaching opportunities have been rapidly increased, which could have impacted on the subsequent validity of the SJT. Thus, we compared the results for the UCAT SJT for 2013 to later years of sittings. Whilst this sensitivity analysis was not sufficiently powered to show a statistically significant difference between the results a trend did seem apparent. That is the raw effect-size for the 2013 UCAT SJT scores seemed somewhat larger than for subsequent years (ORs 0.72 vs 0.91 respectively; see Table S3).

**Table S3.** Sensitivity analysis results comparing the ability of the UCAT SJT scores to predict a student with at least one professionalism lapse for the 2013 test compared to later rounds. Results from the imputed (m=30) and non-imputed data are shown.

| **Testing years**  (Note: HESA rounded values for ‘n’ shown) | **Odds Ratio (OR) (95% CI)** | **p-value** |
| --- | --- | --- |
| **Univariable results from imputed data (m=30)** | | |
| UCAT SJT – 2013 round (n=220) | 0.72 (0.39 to 1.32) | 0.291 |
| UCAT SJT – 2014 onwards (n=470) | 0.91 (0.59 to 1.40) | 0.658 |
| **Results from non-imputed data** | | |
| UCAT SJT – 2013 round (n=200) | 0.72 (0.38 to 1.35) | 0.303 |
| UCAT SJT – 2014 onwards (n=420) | 0.88 (0.57 to 1.36) | 0.559 |
